# Clinical and endoscopic features of primary small bowel lymphoma: a single-center experience from mainland China

**DOI:** 10.3389/fonc.2023.1142133

**Published:** 2023-06-16

**Authors:** Feng-Yu Tian, Jue-Xin Wang, Gang Huang, Wen An, Li-Si Ai, Sui Wang, Pei-Zhu Wang, Yan-Bo Yu, Xiu-Li Zuo, Yan-Qing Li

**Affiliations:** ^1^ Department of Gastroenterology, Qilu Hospital, Shandong University, Jinan, China; ^2^ Shandong Provincial Clinical Research Center for Digestive Disease, Jinan, China; ^3^ Laboratory of Translational Gastroenterology, Qilu Hospital of Shandong University, Jinan, China

**Keywords:** primary small intestinal lymphoma, enteroscopy, gastrointestinal oncology, treatment and prognosis, clinical presentation

## Abstract

**Objective:**

The worldwide incidence of primary small intestinal lymphoma (PSIL) is increasing. However, little is known about the clinical and endoscopic characteristics of this disease. The aim of this study was to investigate the clinical and endoscopic data of patients with PSIL, with the goal of enhancing our understanding of the disease, improving diagnostic accuracy, and facilitating more accurate prognosis estimation.

**Methods:**

Ninety-four patients diagnosed with PSIL were retrospectively studied at Qilu Hospital of Shandong University between 2012 and 2021. The clinical data, enteroscopy findings, treatment modalities, and survival times were collected and analyzed.

**Results:**

Ninety-four patients (52 males) with PSIL were included in this study. The median age of onset was 58.5 years (range: 19-80 years). Diffuse large B-cell lymphoma (n=37) was the most common pathological type. Abdominal pain (n=59) was the most frequent clinical presentation. The ileocecal region (n=32) was the most commonly affected site, and 11.7% of patients had multiple lesions. At the time of diagnosis, the majority of patients (n=68) were in stages I-II. A new endoscopic classification of PSIL was developed, including hypertrophic type, exophytic type, follicular/polypoid type, ulcerative type, and diffusion type. Surgery did not show a significant increase in overall survival; chemotherapy was the most commonly administered treatment. T-cell lymphoma, stages III-IV, “B” symptoms, and ulcerative type were associated with poor prognosis.

**Conclusion:**

This study provides a comprehensive analysis of the clinical and endoscopic features of PSIL in 94 patients. This highlights the importance of considering clinical and endoscopic characteristics for accurate diagnosis and prognosis estimation during small bowel enteroscopy. Early detection and treatment of PSIL is associated with a favorable prognosis. Our findings also suggest that certain risk factors, such as pathological type, “B” symptoms, and endoscopic type, may affect the survival of PSIL patients. These results underscore the need for careful consideration of these factors in the diagnosis and treatment of PSIL.

## 1 Introduction

The gastrointestinal (GI) tract is the predominant site of extranodal non-Hodgkin’s lymphoma, accounting for 30-45% of all cases (1). Primary small intestinal lymphoma (PSIL) is a malignant tumor that originates in the lamina propria and submucosal lymphoid tissue of the small intestine. PSIL occurs infrequently, accounting for 20% to 30% of primary gastrointestinal (GI) lymphomas. Although PSIL is rare, its prevalence has been rising globally (1–4). The majority of primary gastrointestinal lymphomas are classified as non-Hodgkin’s lymphoma (NHL) based on histopathology (5, 6).

Primary small intestinal lymphoma (PSIL) is a rare disease that is often misdiagnosed until serious complications such as obstruction and bleeding develop. Although progress has been made in the diagnosis and treatment of gastric lymphomas, PSIL remains poorly characterized, and little is known about its clinical, enteroscopic, and pathological features. Furthermore, the optimal treatment for PSIL remains controversial, and the prognosis is unsatisfactory. The small bowel was previously inaccessible to endoscopists because of its depth, length, and complicated loops, limiting the possibility of large-scale studies on PSIL. Therefore, more research is needed to improve the diagnosis and management of PSIL (7, 8).

The introduction of balloon-assisted enteroscopy, including double-balloon enteroscopy (DBE) and single-balloon enteroscopy (SBE), has led to an increased number of patients being diagnosed with primary small intestinal lymphoma (PSIL) through endoscopic examination. However, the lack of information on the typical endoscopic features of PSIL makes it difficult to accurately diagnose the disease. Therefore, the purpose of this study was to evaluate the clinical and endoscopic features, management, and prognosis of PSIL patients diagnosed at Qilu Hospital of Shandong University over a period of approximately ten years to enhance our understanding of this disease.

## 2 Methods

### 2.1 Patients

From January 2012 to August 2021, a retrospective review of medical records and pathological data was conducted on 94 individuals with small intestine lymphoma who were identified through a database search at the Department of Pathology and the Digestive Endoscopic Center at Qilu Hospital of Shandong University. The patients’ information was anonymized prior to analysis. Histopathological diagnosis was based on the WHO classification (9, 10) and performed through morphologic and immunophenotypic analyses of endoscopically biopsied or surgically resected specimens. Patients were included in the study based on the definition of primary gastrointestinal non-Hodgkin’s lymphoma according to Lewin et al. (11). Those patients who presented with second malignancies or without follow-up information were excluded.

The diagnostic workup included a detailed medical history and physical examination, complete blood cell count, serum chemistry, abdominal ultrasonography, chest/abdomen/pelvis computed tomography (CT) scan, bone marrow aspiration or biopsy, and multiple endoscopic biopsies. If feasible, positron emission tomography (PET) imaging was performed in some patients.

### 2.2 Stage

Musshoff’s variation of the Ann Arbor method for gastrointestinal lymphoma was utilized for staging (12), and the imaging modality for clinical staging was CT or PET.

### 2.3 Endoscopic features

We updated the endoscopic classification system for small intestine lymphoma based on previous studies (5, 13–16), and two competent endoscopists assessed the endoscopic pictures and classified the endoscopic findings into five categories (1): hypertrophic type (2), exophytic tumor type (3), follicular/polypoid type (4), ulcerative type, and (5) diffusion type.

### 2.4 Follow-up and statistical analysis

The survival of patients with small intestinal lymphoma was evaluated by the number of days. Overall survival (OS) was calculated from the date of diagnosis until the date of death or the final follow-up visit.

All analyses were performed using SPSS Statistics 23.0 software (SPSS Inc., Chicago, IL, USA). Normal continuous variables were reported as the means ± standard deviations, and significant differences were evaluated using Student’s t test. Nonnormally distributed continuous variables were reported as medians (25th percentile - 75th percentile). Significant differences were evaluated using the Mann−Whitney U test. During the nonparametric tests, proportions were compared using the chi-square test. In cases where the expected values were insufficient to meet the requirements of the chi-square test, Fisher’s exact test was employed. When conducting pairwise comparisons between multiple groups of categorical variables, the Bonferroni correction method was employed, or they were performed by using *post hoc* testing and observing standardized residuals to determine the significance of the differences. Overall survival was estimated using the life table and Kaplan−Meier product-limit method, and the values were compared using the log-rank test. Differences were considered significant when the *P* values were less than 0.05.

## 3 Results

### 3.1 Clinical features

**Figure 1:**
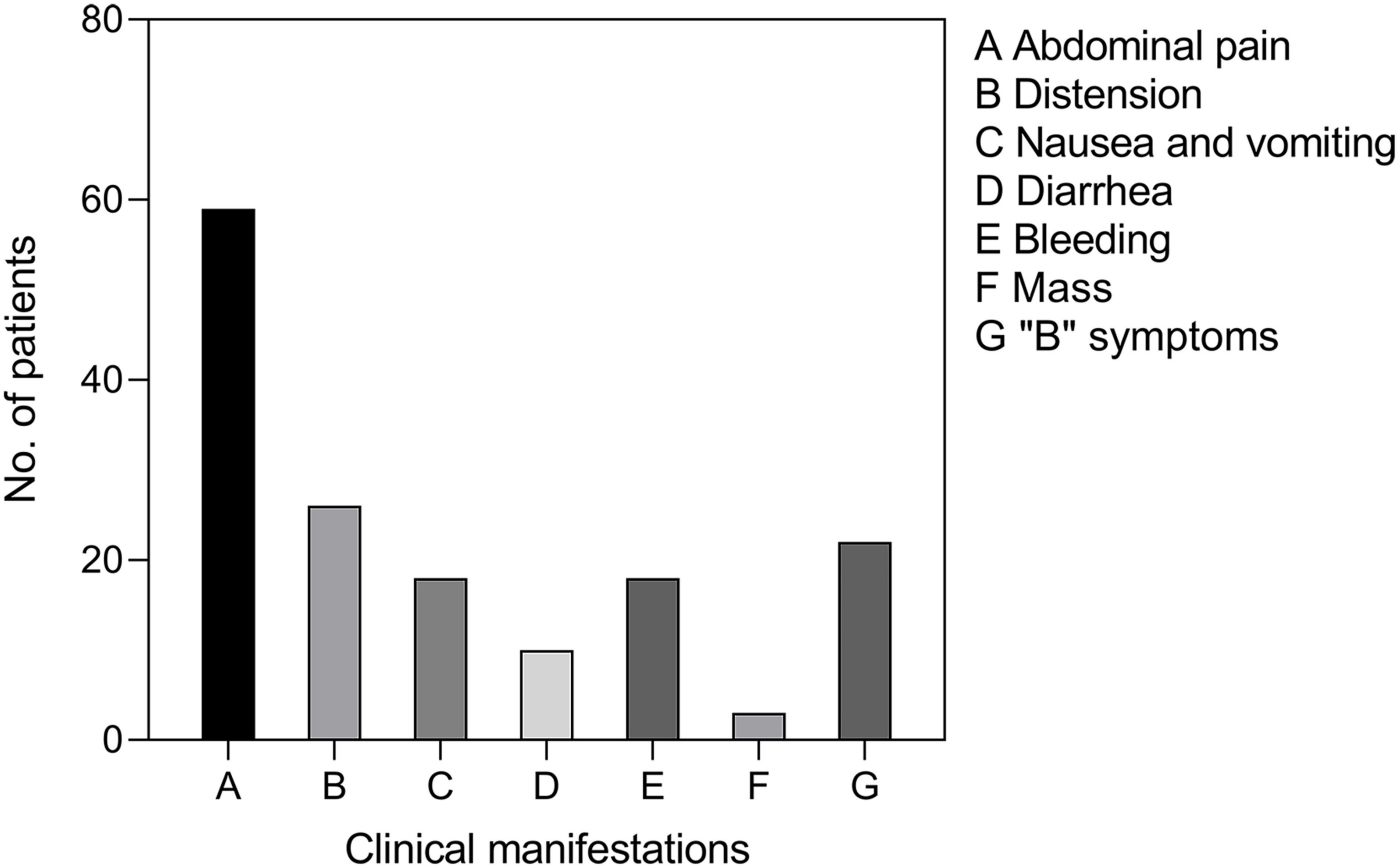
Clinical manifestations of PSIL. Abdominal pain was the most common symptom, and some patients had two or more symptoms. “B” symptoms: symptoms of fever, night sweats, and weight loss.

From January 2012 to August 2021, a total of 94 patients diagnosed with primary small intestinal lymphoma were included in this analysis. **Table 1** summarizes the clinical characteristics of these patients. The median age of the cohort was 58.50 (48.00-66.25) years, with a gender distribution of 52 males and 42 females, and an age range of 19 to 80 years. The most common symptom was abdominal pain (n = 59), followed by abdominal distension (n = 26), nausea and vomiting (n = 18), hematemesis or hematochezia (n = 18), diarrhea (n = 10), and mass (n = 3). Twenty-two individuals exhibited “B” symptoms (fever, nocturnal sweats, and weight loss) (**Figure 1**). Of all patients, 19 (20.2%) acquired illness in the duodenum, 17 (18.1%) in the jejunum, 15 (15.9%) in the ileum, 32 (34.0%) in the ileocecal region, and 11 (11.7%) in more than one location.

**Table 1:** Demographic and Clinical Characteristics of Primary Small Intestinal Lymphoma.

Characteristics	Value
Median age (range), years	58.50(48.00-66.25)
Gender(M), n(%)	52(55.3)
Symptoms, n(%)	
Abdominal pain	59(66.3)
Distension	26(29.2)
Nausea and vomiting	18(20.2)
Diarrhea	10(11.2)
Hematemesis, hematochezia	18(20.2)
Mass	3(3.4)
“B” symptoms	22(24.7)
Site, n(%)	
Duodenum	19(20.2)
Jejunum	17(18.1)
Ileum	15(16.0)
Ileocecum	32(34.0)
Multiple	11(11.7)
Stage, n(%)	
I-II	68(74.7)
III-IV	23(25.3)
Laboratory index, n(%)	
HGB decreased	36(41.9)
LDH increased	28(32.6)
Low ALB	45(52.3)
ADA increased	19(23.5)
Imaging features(CT), n(%)	
Thickening	50(64.9)
Occupying	16(20.8)
Lymph node enlargement	54(74.0)
5-year OS(%)	62.00

HGB, Hemoglobin; LDH, Lactate dehydrogenase; ALB, Albumin; ADA, Adenosine deaminase.

Among the various clinical variables studied, a higher number of patients exhibited abnormalities in certain indicators such as hemoglobin (HGB), albumin (ALB), lactate dehydrogenase (LDH), and adenosine deaminase (ADA). However, complete data for these specific indicators were not available for all patients. Among the 86 patients with available data, 38.3% had lower HGB levels, 47.9% had lower ALB levels, and 29.8% had higher LDH levels than the standard range. In the subset of 81 patients with available ADA data, 20.2% had ADA levels above the standard range. Additionally, 35 (40.7%) patients had two or more abnormal laboratory indicators (**Table 1**).

During computed tomography (CT) examination, intestinal wall thickening of varying degrees, space-occupying lesions in the small intestine, and celiac lymph node enlargement were observed in the patients. Sixty-eight (74.7%) patients had stage I-II disease, and 23 (25.3%) had stage III-IV disease (**Table 1**).

### 3.2 Histopathology

**Figure 2:**
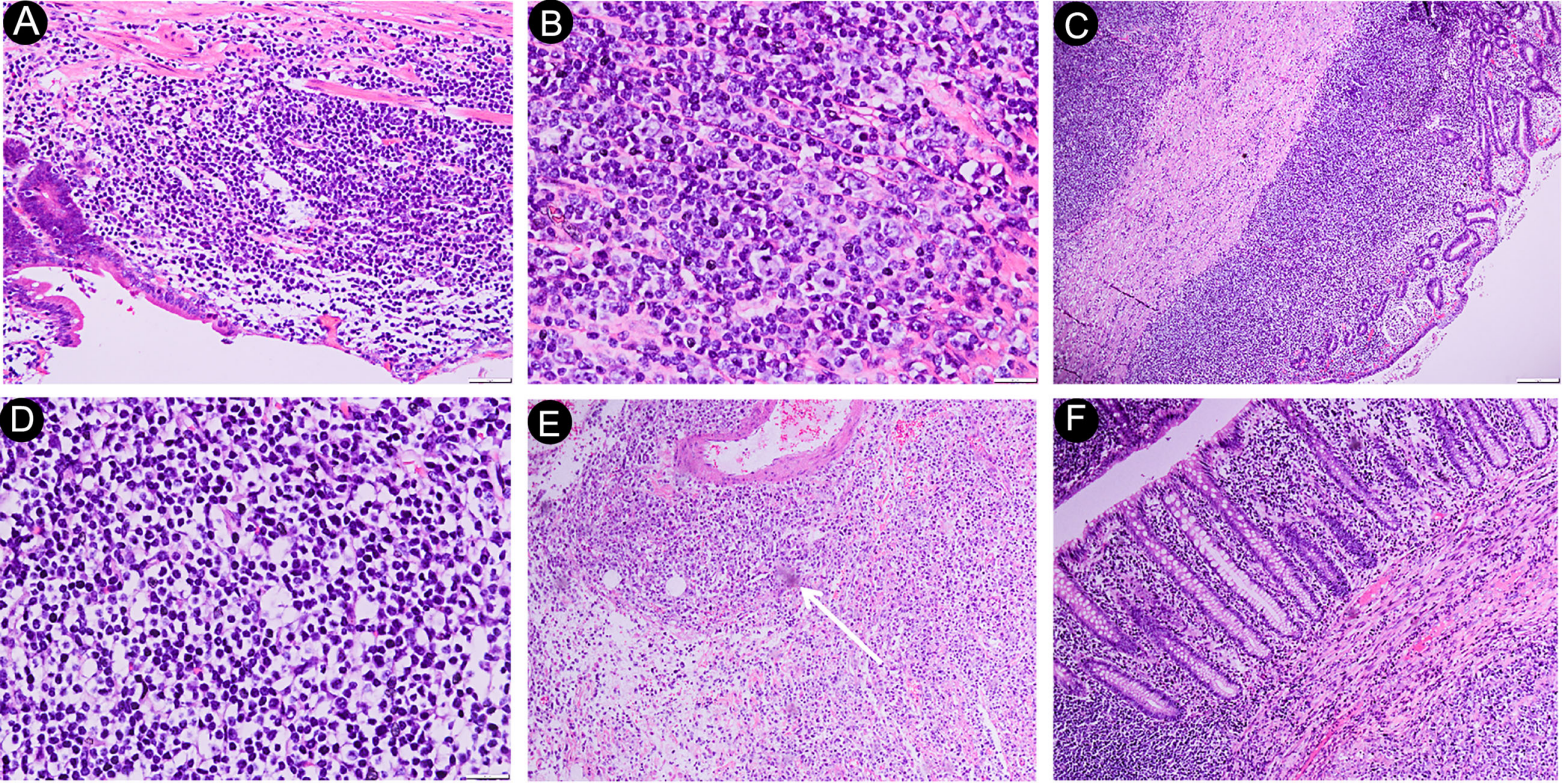
Histological features of PSIL. DLBCL of the ileum, photomicrograph (original magnification ×200, 400; H-E stain) of the specimen shows diffuse large lymphocytes (> 2.5-times larger than normal cells) infiltrating into the intestinal epithelium; no germinal centers were observed; some lymphocytes had nucleoli; lymphoepithelial lesions; part of the muscular layer had been destroyed **(A, B)**. MALT of the jejunum, photomicrograph (original magnification ×40, 400; H-E stain). Histologically, extensive infiltration of normal-sized lymphocytes was observed throughout the corresponding intestinal wall and, occasionally, into the subserosa **(C, D)**. Follicular lymphoma of the duodenum. The photomicrograph (original magnification ×40; H-E stain) shows the presence of individual submucosal lymphoid follicles (arrow), and no apoptotic appearance was observed **(E)**. Monomorphic epitheliotropic intestinal T-cell lymphoma of the jejunum. The photomicrograph (original magnification, ×40; H-E stain) shows small- to focally medium-sized lymphocytes diffusely infiltrating into the intestinal epithelium. The nuclei are oval-shaped or distorted **(F)**. DLBCL, diffuse large B-cell lymphoma; MALT, mucosa-associated lymphoid tissue lymphoma.

Diffuse large B-cell lymphoma (DLBCL), which comprises lymphocytes invading the intestinal epithelium (**Figures 2 A, B**), was the most prevalent subtype (37 patients). Mucosa-associated lymphoid tissue lymphoma (MALT lymphoma) was the second most common pathological type (27 patients), and it is characterized by significant infiltration of lymphocytes of normal size throughout the intestinal wall (**Figures 2C, D**). Eleven people were diagnosed with follicular lymphoma (FL). The formation of multiple or single submucosal lymphoid follicles was the pathological characteristic of the biopsy tissue (**Figure 2E**). Monomorphic epitheliotropic T-cell lymphoma (MEITL) and enteropathy-associated T-cell lymphoma (EATL) are primary intestinal lymphomas derived from intraepithelial lymphocytes, which are often characterized by diffuse infiltration of focally medium-sized lymphocytes in the intestinal epithelium (**Figure 2F**). In our study, we identified 2 patients with EATL and 3 patients with MEITL. In addition, there were 3 patients with mantle cell lymphoma (MCL), 3 patients with peripheral T-cell lymphoma (PTCL), and 2 patients with extranodal NK/T-cell lymphoma (ENKL).

**Figure 3:**
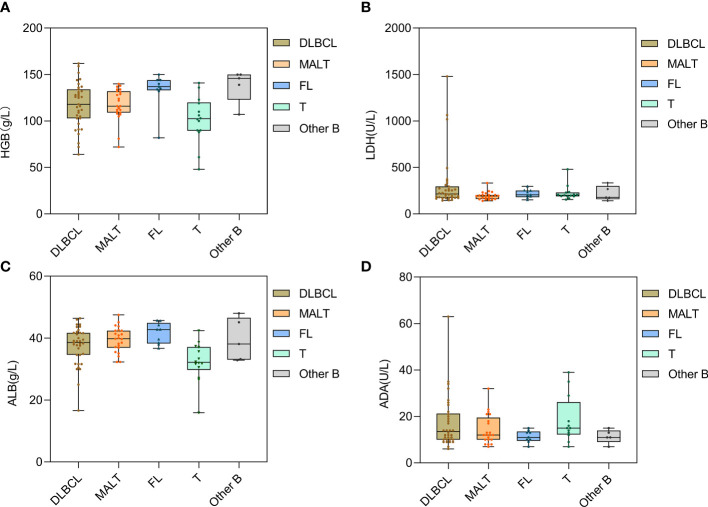
Distribution of laboratory indexes of PSIL in different pathological types. Patients with T-cell lymphoma showed lower HGB and ALB levels **(A, C)**. Patients with DLBCL had large fluctuations in LDH and ADA levels **(B, D)**. DLBCL, diffuse large B-cell lymphoma; MALT, mucosa-associated lymphoid tissue lymphoma; FL, follicular lymphoma; T, T-cell lymphoma; Other B, mantle cell lymphoma and other small B-cell lymphomas. HGB, hemoglobin; LDH, lactate dehydrogenase; ALB, albumin; ADA, adenosine deaminase.

We further divided all patients into five pathological subgroups (DLBCL, MALT lymphoma, FL, T-cell lymphoma, and other B-cell lymphoma) to examine the major laboratory indicators and found that the LDH and ADA levels fluctuated significantly in patients with DLBCL. The HGB and ALB levels were lower in T-cell lymphoma and were relatively higher in patients with FL (**Figure 3**).

### 3.3 Enteroscopic features and correlation analysis

**Figure 4:**
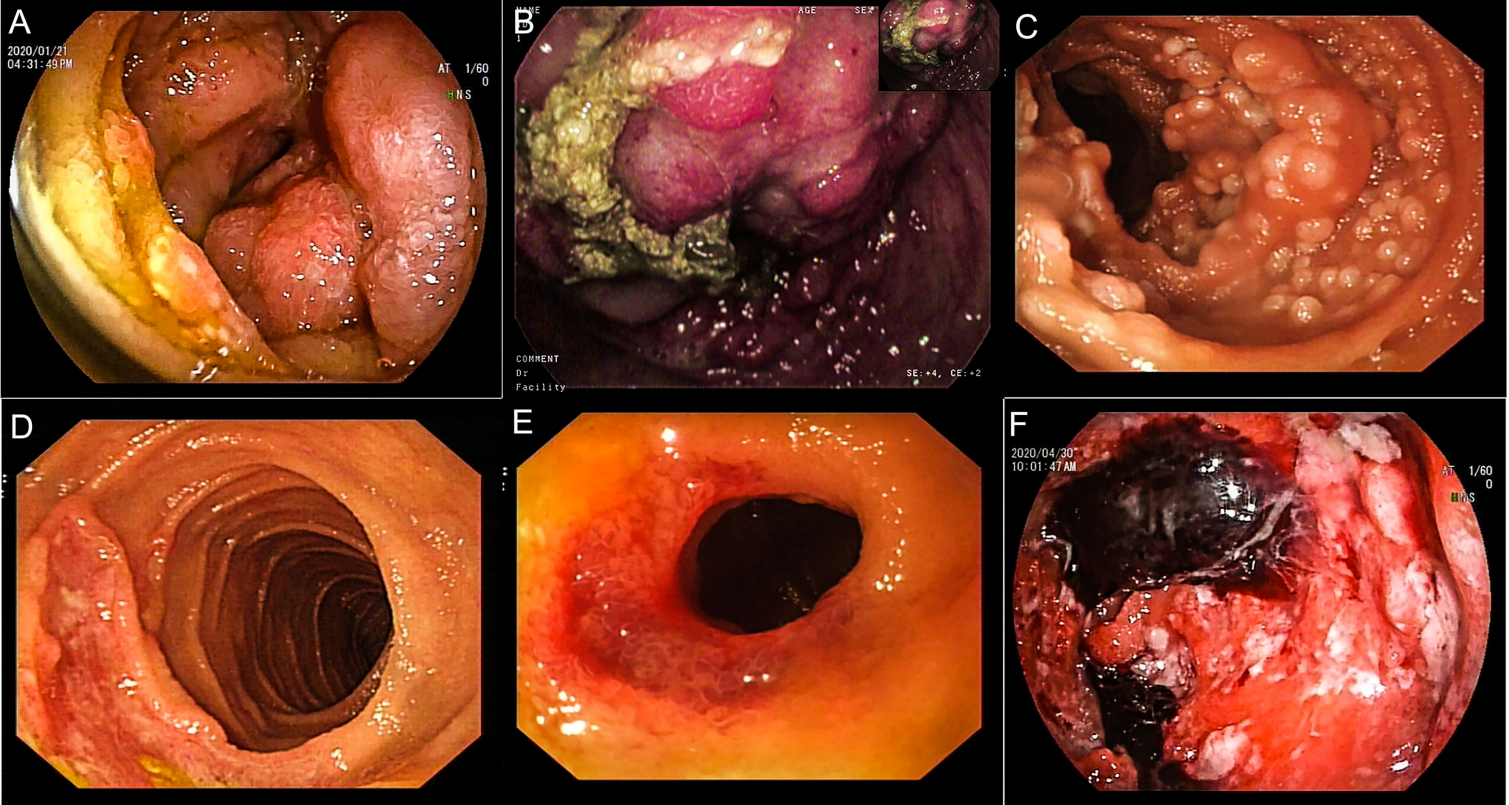
Endoscopic findings of patients with PSIL. Hypertrophic type, irregular mucosal eminence around the duodenum, pathologically confirmed as T-cell lymphoma **(A)**. Exophytic type, ileocecum showed a huge protuberant lesion, surface hyperemia, edema and erosion, pathologically confirmed as MCL **(B)**. Follicular/polypoid type, follicular mucosal eminence diffusely distributed in the proximal small intestine, and pathologically confirmed as FL **(C)**. Ulcerative type, irregular ulcer spots on the ileocecal area, pathologically confirmed as MALT lymphoma **(D)**. Diffusion type, lower jejunum circumferential stenosis, surface ulceration, blood scab attachment, pathology confirmed as MALT **(E)**. Diffusion type, diffuse mucosal hyperemia erosion in the middle part of jejunum, covered with mucus, old blood clots, a rigid wall, a narrow lumen, and DLBCL was confirmed by pathology **(F)**. DLBCL, diffuse large B-cell lymphoma; MALT, mucosa-associated lymphoid tissue lymphoma; FL, follicular lymphoma; MCL, mantle cell lymphoma.

During hospitalization, all patients underwent an enteroscopy examination. The endoscopic features were divided into the following groups: 16 patients had a hypertrophic type, characterized by edematous and irregular folds resulting in intestinal strictures that remained after maximum insufflation; 23 patients had an exophytic type, characterized by tumor-like lesions such as a large, friable, nodular mass with signs of hemorrhage, surface erosion, or moss cover; 17 patients had a follicular/polypoid type, characterized by multiple lymphoid follicular eminences or multiple polypoid lesions; 19 patients had an ulcerative type, characterized by single ulceration or multiple ulcerative lesions; and 19 patients had a diffuse type, characterized by mucosal erosion, punctate or patchy bleeding, mucosal rough hyperplasia, and other endoscopic manifestations. Among these patients, 4 had two or more endoscopic features simultaneously. For statistical analysis purposes, we selected the main endoscopic feature for each patient. All endoscopic characteristics are shown in **Figure 4**.

**Figure 5:**
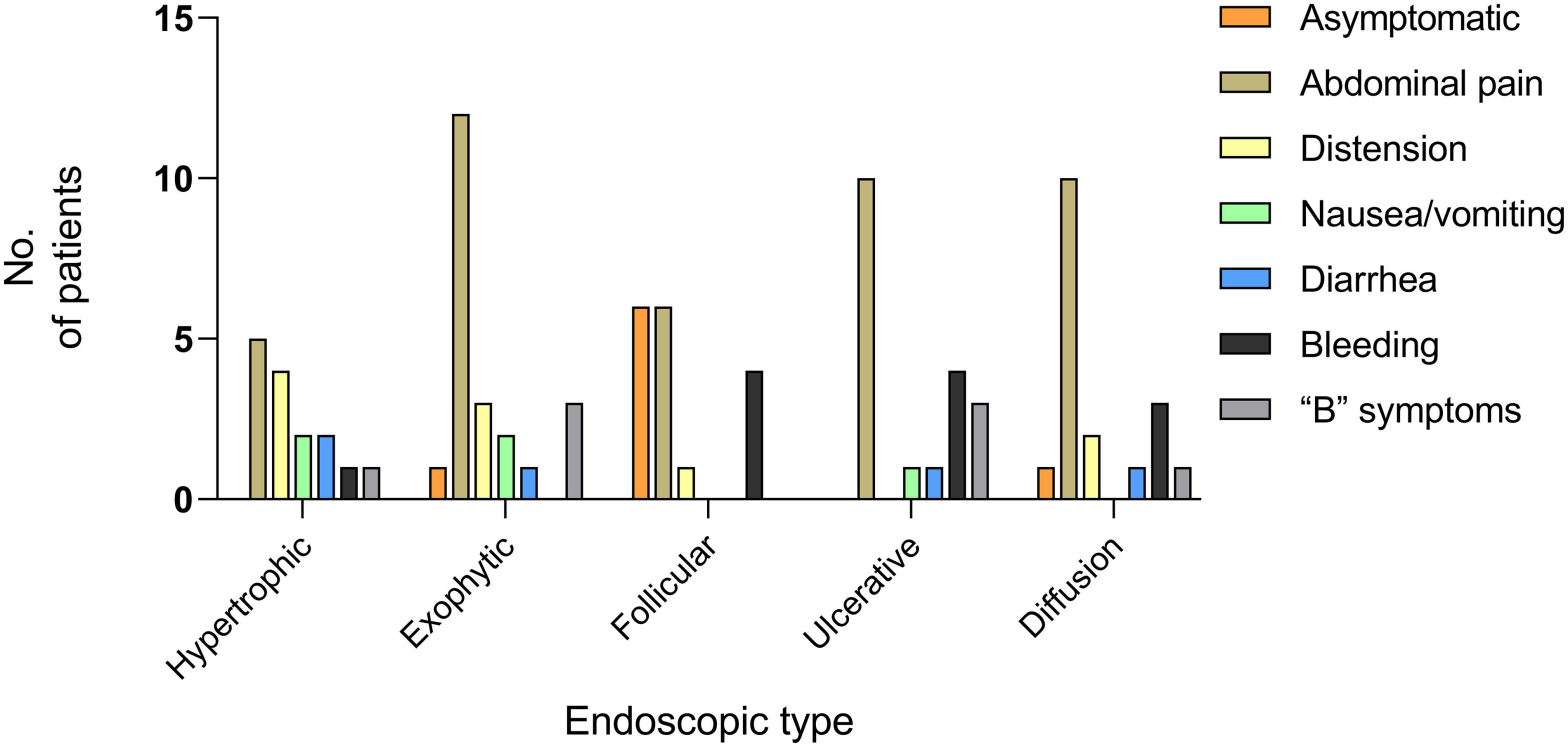
Clinical manifestations of PSIL in five endoscopic types. Abdominal pain emerged as the predominant symptom across all endoscopic types. Among the asymptomatic patients, 75% exhibited the follicular/polypoid type. The clinical symptoms in the data were the main symptoms of the patients. “B” symptoms: symptoms of fever, night sweats, and weight loss.

Abdominal pain was the most prevalent symptom across all endoscopic types. The overall distribution of other clinical symptoms among the different endoscopic types is detailed in **Figure 5**. The follicular/polypoid form was identified in 75% of asymptomatic patients. Bleeding (including hematemesis and hematochezia) was observed in 33.3% of follicular/polypoid lesions and 41.7% of ulcerative lesions. “B” symptoms were observed in 37.5% of exophytic lesions and 37.5% of ulcerative lesions. Based on Fisher’s exact test and subsequent *post hoc* testing, it was observed that in FL, the actual value of asymptomatic patients exceeded the expected value, with a standardized residual of 3.7. This suggests that patients with FL are more likely to present without abdominal and systemic symptoms, and the difference is significant (**Supplementary Table S1**).

**Figure 6:**
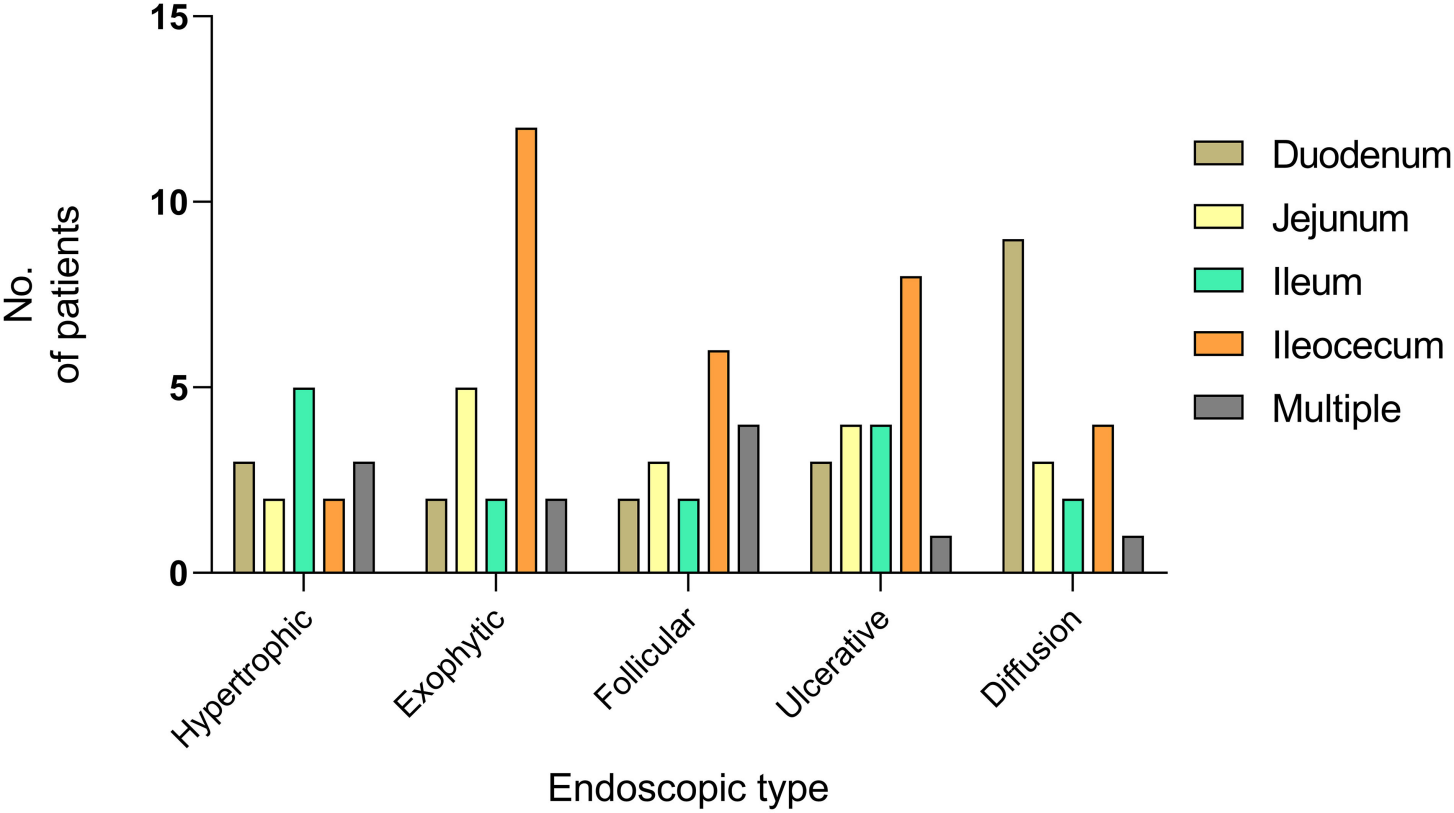
Onset sites of PSIL in different endoscopic types. Fifty-two percent of the exophytic type and 40% of the ulcerative type occurred in the ileocecal region, and 47.4% of patients with the diffusion type developed the disease in duodenum.

A total of 52.2% of the exophytic type originated in the ileocecal area, whereas 47.4% of the diffusion type originated in the duodenum (**Figure 6**). As visualized by imaging analysis, lymph node enlargement was observed in 85.0% of the exophytic type cases, 91.7% of the follicular/polypoid type, and 71.4% of the ulcerative type. However, it was reported in only 50.0% of the hypertrophic type cases and 52.9% of the diffusion type. The differences among the groups were statistically significant (*P* = 0.043). No significant differences were obtained in thickening and occupying among the subtypes of endoscopic features.

A histopathological diagnosis was performed on all patients, and the associations between endoscopic and pathological features were determined using Fisher’s exact test and the Bonferroni correction for multiple group comparisons. The results revealed a statistically significant difference between the groups (*P* < 0.05). Specifically, within the DLBCL subgroup, the exophytic type demonstrated a higher prevalence than the follicular/polypoid type. In the MALT subgroup, the diffusion type exhibited a greater occurrence than the exophytic type. Furthermore, in the FL subgroup, the follicular/polypoid type was more frequently observed than other types. No significant differences were observed among the different endoscopic types within the T-cell lymphoma subgroup (**Table 2**).

**Table 2:** Correlation between Endoscopic Features and Pathology of Primary Small Intestinal Lymphoma.

Endoscopic features	Pathological type				*P* value^1^
	DLBCL, n(%)	MALT, n(%)	FL, n(%)	T, n(%)	
Hypertrophic type	8(21.6)	6(22.2)	1(9.1)	1(7.1)	<0.01
Exophytic type	14(37.8)	1(3.7)	0(0.0)	6(42.9)	
Follicular/polypoid type	2(5.4)	3(11.1)	9(81.8)	0(0.0)	
Ulcerative type	7(18.9)	7(25.9)	1(9.1)	4(28.6)	
Diffusion type	6(16.2)	10(37.0)	0(0.0)	3(21.4)	
Total	37	27	11	14	

^1^The Fisher’s exact test indicated a significant difference between groups.

DLBCL, Diffuse large B-cell lymphoma; MALT, Mucosa-associated lymphoid tissue lymphoma; FL, Follicular lymphoma; T: T-cell lymphoma.

### 3.4 Clinical correlation among the subgroups

To investigate the association between clinical and endoscopic characteristics, patients were divided into four categories based on sex (male and female), age (≥ 65, < 65), stage (stages I-II and stages III-IV), and pathological type (B-cell and T-cell lymphoma).

There were significant variations in HGB between the sexes (*P* < 0.01). Age (*P* = 0.233), onset location (*P* = 0.412), histological type (*P* = 0.131), endoscopic characteristics (*P* = 0.812), clinical symptoms (*P* = 0.372), LDH (*P* = 0.258), ALB (*P* = 0.818), and ADA (*P* = 0.913) were not significantly different between male and female patients. (**Supplementary Table S2**).

The analysis of patients in distinct age categories revealed statistically significant variations in pathological type distribution (*P* = 0.048). According to the statistical data, FL was more prevalent among patients under 65 years of age. The onset site (*P* = 0.088), endoscopic features (*P* = 0.463), clinical symptoms (*P* = 0.294), hemoglobin (*P* = 0.970), LDH (*P* = 0.493), ALB (*P* = 0.111), and ADA (*P* = 0.744) were not significantly different between the age groups (**Supplementary Table S3**).

Compared with patients in stages I-II, patients in stages III-IV showed lower HGB (*P* = 0.026) and ALB (*P* < 0.01) levels, as well as elevated LDH (*P* < 0.01) and ADA (*P* < 0.01) levels. Ages, genders, and histopathologic types of patients in stages I-II and stages III-IV did not vary significantly (*P* > 0.05). (**Supplementary Table S4**).

The clinical characteristics of B-cell lymphoma and T-cell lymphoma were compared. Based on the pairwise comparisons between the groups, the incidence rate of “B” symptoms was higher in T-cell lymphoma than in B-cell lymphoma, indicating a significant difference (*P* = 0.038). In addition, T-cell lymphoma was associated with decreased levels of HGB and ALB (*P* < 0.01). No discernible differences were observed between B-cell and T-cell lymphomas in terms of endoscopic characteristics (*P* = 0.130), imaging characteristics such as thickening (*P* = 0.500), occupying (*P* = 0.549), or lymph node enlargement (*P* = 0.799), treatment strategy (*P* = 0.633), or laboratory indicators such as LDH (*P* = 0.797) and ADA (*P* = 0.136) (**Supplementary Table S5**).

### 3.5 Treatment and prognosis

Chemotherapy was the primary treatment modality used for 44 patients, while 17 patients chose to undergo surgery before receiving chemotherapy. Among these, four T-cell lymphoma patients died as a result of postoperative chemotherapy complications, such as intestinal obstruction, perforation, gastrointestinal bleeding, and abdominal infection. At the most recent follow-up, an additional 13 patients achieved complete remission (CR) and were still alive. Primary surgery was performed as an emergency measure for four patients experiencing obstruction (one patient), perforation (one patient), and severe abdominal pain (two patients). Of these, three patients (two with DLBCL and one with FL) had complete remission without relapse, while the fourth patient with MCL required emergency surgery due to severe abdominal pain after chemotherapy and died a month later. Five DLBCL patients and two MALT lymphoma patients underwent surgery alone, with two DLBCL patients lost to follow-up after discharge. The remaining three DLBCL patients and two MALT lymphoma patients achieved complete remission.

A larger proportion of patients with MALT lymphoma (15 out of 25) and T-cell lymphoma (8 out of 15) received chemotherapy alone, while a greater percentage of DLBCL patients (12 out of 35) received chemotherapy in combination with surgery. Only six patients with stage I DLBCL received surgery alone. Four patients with FL and seven patients with MALT lymphoma received conservative management, which included but was not limited to appropriate nutritional support, psychological support, physical therapy, and symptom control medication (**Table 3**).

**Table 3:** Treatment According to Pathology in Patients with Primary Small Intestinal Lymphoma.

Endoscopic features	No. of patients	Surgery + chemotherapy	Chemotherapy	Surgery	Conservative management
Hypertrophic type	14	4	4	4	2
Exophytic type	22	7	9	4	2
Follicular/polypoid type	16	2	9	0	5
Ulcerative type	20	3	13	0	4
Diffusion type	17	3	10	0	4

^1^Selective surgical treatment before chemotherapy or surgical treatment due to complications during chemotherapy, one of the patients with MCL underwent surgical treatment because of serious complications half a year after chemotherapy, which does not belong to this category.

^2^ Conservative management strategies encompass a wide range of interventions, including but not limited to appropriate nutritional support, psychological support, physical therapy, and symptom control medication.

DLBCL, Diffuse large B-cell lymphoma; MALT, Mucosa-associated lymphoid tissue lymphoma; FL, Follicular lymphoma; MCL, Mantle cell lymphoma; T, T-cell lymphoma.

Eight of 14 patients with the hypertrophic type and 11 of 22 patients with the exophytic type underwent surgery (**Table 4**). There were no discernible differences in survival rates between the surgical and nonsurgical groups for any endoscopic feature category.

**Table 4:** Treatment According to the Endoscopic Type in Patients with Primary Small Intestinal Lymphoma.

Endoscopic features	No. of patients	Surgery + chemotherapy	Chemotherapy	Surgery	Conservative management
Hypertrophic type	14	4	4	4	2
Exophytic type	22	7	9	4	2
Follicular/polypoid type	16	2	9	0	5
Ulcerative type	20	3	13	0	4
Diffusion type	17	3	10	0	4

Conservative management strategies encompass a wide range of interventions, including but not limited to appropriate nutritional support, psychological support, physical therapy, and symptom control medication.

**Figure 7:**
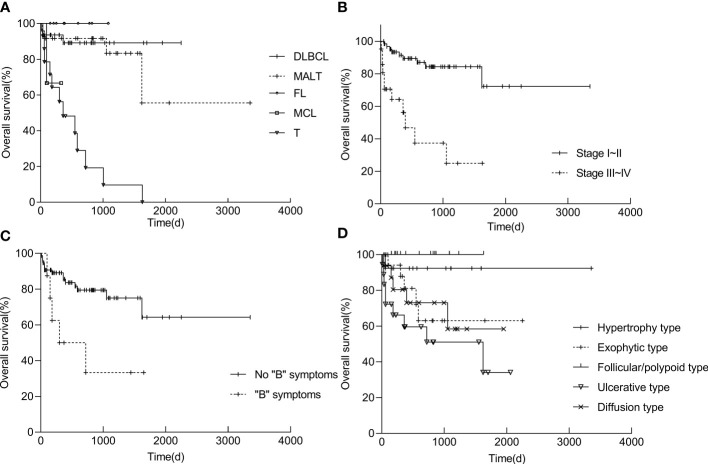
Kaplan−Meier survival curves of PSIL in pathological group **(A)**, stage group **(B)**, symptom group **(C)** and endoscopic group **(D)**. Statistics of the patient survival rates were determined by 30-3353 days of follow-up. The life table and Kaplan−Meier product−limit method were used, and values were compared using the log rank test. Differences were considered significant when the *P* values were less than 0.05. DLBCL, diffuse large B-cell lymphoma; MALT, mucosa-associated lymphoid tissue lymphoma; FL, follicular lymphoma; MCL, mantle cell lymphoma; T, T-cell lymphoma; “B” symptoms: symptoms of fever, night sweats, and weight loss.

Survival analyses were performed in 84 patients (of whom 10 were lost to follow-up), and the follow-up period ranging from 30 to 3353 d (mean, 692.6 d). Overall survival (OS) at five years was 62.0% (**Table 5**). The survival rate of patients in stages I-II was significantly higher than that of patients in stages III-IV (*P* < 0.01). T-cell lymphoma (*P* < 0.01) and “B” symptoms (P = 0.014) were related to a worse prognosis. Patients with ulcerative type had a lower OS, whereas patients with follicular/polypoid type had a better survival rate (**Figure 7**). Other variables, including sex, age, illness location, imaging characteristics (CT), and high LDH, had no influence on the survival rate (**Table 5**).

**Table 5:** Statistical Results of Survival and Prognosis.

Characteristics	n	5-year OS	*P* value
Gender			
Male	46	57.0	0.679
Female	38	74.0	
Age			
<65	45	69.0	0.485
≥65	39	58.0	
Site of origin			
Duodenum	15	32.0	0.410
Jejunum	16	66.0	
Ileum	13	91.0	
Ileocecum	29	72.0	
Multiple	11	65.0	
Symptom			
“B” symptoms	8	36.0	0.014
No “B” symptoms	76	65.0	
Stage			
I-II	64	73.0 .0	<0.01
III-IV	20	27.0	
Pathology			
DLBCL	31	89.0	<0.01
MALT	24	63.0	
FL	10	100.0	
MCL	3	67.0	
T	14	0.0	
Endoscopic type			
Hypertrophic type	14	92.0	0.016
Exophytic type	20	66.0	
Follicle/polyp type	16	100.0	
Ulcerative type	18	34.0	
Diffusion type	16	61.0	
LDH			
Elevated	25	65.0	0.293
Normal	54	72.0	
Therapy			
Surgery + chemotherapy	19	88.0	0.716
Chemotherapy alone	44	53.0	
Surgery alone	6	80.0	
Conservative management	15	71.0	

Accessed by the log-rank test; B, B-cell lymphoma; T, T-cell lymphoma; LDH, Lactate dehydrogenase.

Conservative management strategies encompass a wide range of interventions, including but not limited to pain management, appropriate nutritional support, psychological support, physical therapy, rehabilitation therapy, and symptom control medication.

## 4 Discussion

The diversity of PSIL in terms of patient features, stage, histologic subtypes, clinical symptoms, therapy, and prognosis has been widely acknowledged. While primary gastrointestinal lymphoma, particularly primary gastric lymphoma, has been the subject of numerous studies (1, 17–19), PSIL has received relatively little attention, with few studies conducted in mainland China (1, 20–22). In 2007, L. Yin et al. performed a retrospective study on 34 PSIL patients, which represents one of the few studies on this topic in China (7). In this study, we present the largest retrospective cohort of PSIL patients reported to date, providing a comprehensive analysis of clinical and histological characteristics, establishing an endoscopic classification, and conducting a survival analysis to investigate the long-term survival outcomes of PSIL patients.

Primary gastrointestinal lymphoma has been reported to have a male predominance and an average age of onset of ≥50 years, especially in the ileocecal region, with a male-to-female ratio up to 2.7:1 (11, 23–32). In our research, the average age was 56.4 years, and there was only a slight male preponderance for the entire small intestine (male to female ratio of 1.2:1) and the ileocecal region (male to female ratio of 1.3:1), which is lower than that reported in previous studies.

PSIL may manifest clinically in a variety of ways, with pain being the most prevalent symptom in all instances, followed by obstructive symptoms such as distension, nausea, and vomiting, as well as bleeding and diarrhea. The occurrence of “B” symptoms varies from 11.9% to 25% (7, 23, 33, 34).

Regarding illness location, we noticed that 34.1% of PSIL occurred in the ileocecal area, confirming prior findings that the ileocecal region is the most prevalent site for PSIL (1, 6, 23, 35). Koch P et al. noted that ileocecal lymphoma may indicate a good prognosis (23). In our series, no significant differences were observed in the outcomes among different disease locations. This overall improvement in prognosis may be attributed to the advancements made in the detection and treatment of PSIL.

Most authors have utilized the Ann Arbor classification with revisions by Musshoff or the Lugano staging method to determine the extent of GI lymphoma (12, 36). With the exception of the research by Wang W et al. (37), which revealed more patients with stage III-IV (72%) disease, most patients presented with early-stage (I-II) disease (17, 22, 29, 38). In our study, the majority of patients (74.4%) were classified as stages I-II, while 25.3% were classified as stages III-IV. Our findings confirm that early-stage disease (stages I-II) is associated with higher 5-year overall survival rates (73.0% for stages I-II vs. 27.0% for stages III-IV), consistent with previous reports (20, 21, 23, 39).

DLBCL and MALT lymphoma were the predominant pathological subtypes in our cohort, consistent with prior research (11, 17, 26, 40–42). MALT lymphoma is commonly found in the stomach, possibly due to the association with *Helicobacter pylori infection* as a significant risk factor. The B-cell phenotype represents more than 90% of gastrointestinal lymphomas (43, 44). In contrast to European countries, China appears to have a higher incidence of T-cell lymphoma in primary gastrointestinal lymphoma (1, 18, 23, 37). The small intestine is more susceptible to T-cell lymphoma, accounting for 16.6%-25.0% of cases (23, 29). In the current research, T and B lymphocyte phenotypes accounted for 14.9% and 85.1% of small intestine cases, respectively.

Based upon the presenting symptoms, investigations are usually requested in the diagnosis of PSIL, including endoscopy, CT, or PET (45). In our study, the CT findings of PSIL included wall thickening, lymph node hypertrophy, and lymph node occupation. These signs are indicative of the proliferation and lymphatic metastasis of intestinal lymphoma, which have important implications for the CT diagnosis of small intestinal lymphoma. Both SBE and DBE offer the benefit of tissue diagnosis during examination (46–48). A study of 13 patients with FL found that DBE indicated multifocal FL lesions in the jejunum and/or ileum in all patients, while abdominal CT and PET only detected GI tract involvement in one and two patients, respectively (49). In our study of 94 patients with PSIL, 88.3% obtained histological diagnoses through enteroscopy (including DBE and SBE).

Asyia Ahmad et al. initially classified gastrointestinal MALT lymphoma into the exogenous type, hypertrophic type, and ulcerative type based on endoscopy findings (5). In 2010, Angelo Zullo updated this classification and proposed a modified version, including the ulcerative type, exophytic type, hypertrophic type, petechial hemorrhage type, and normal/hyperemic type (15, 16). Another study on NK/T-cell lymphoma in the gastrointestinal tract identified four endoscopic types: superficial/erosive, ulcerative, ulceroinfiltrative, and infiltrative (13). In our study, we developed a novel categorization of five endoscopic subtypes of PSIL based on previous research. In this study, we comprehensively investigated various types of lymphomas. To ensure the inclusiveness of the new endoscopic classification system for these lymphoma types, we made selective updates to the existing endoscopic classifications reported in prior studies. Notably, in the case of follicular lymphoma (FL), a predominant presentation of multiple lymphoid follicles in the intestinal wall was observed during endoscopic examinations. To emphasize its distinctive features, we introduced a novel endoscopic phenotype termed the follicular/polypoid type. Furthermore, certain patients exhibited benign alterations such as superficial erosion or mild bleeding, which had been previously categorized as the normal/hyperemic type or superficial/erosive type in earlier investigations. By combining the endoscopic characteristics of both types, we defined this entity as the diffusion type. In DLBCL, exophytic endoscopic manifestations were significantly more common than the follicular/polypoid type, while in MALT lymphoma, the diffusion type was notably more prevalent than the exophytic type. This may be because exophytic masses tend to develop from particular pathogenic types, such as DLBCL. However, the majority of low-grade malignant GI lymphoma manifests with diffuse submucosal tumor infiltration (5, 50). Given that the follicular/polypoid type of FL is the most common and that the diagnosis under endoscopy was made in the majority of patients before nonspecific abdominal symptoms, we posit that precise recognition of endoscopic features holds considerable value in facilitating early detection and intervention of FL.

The most prevalent symptom across all the endoscopic types was abdominal discomfort. The fact that the majority of asymptomatic patients had follicular/polypoid endoscopic characteristics suggests that patients with the follicular/polypoid type may receive their pathology diagnosis by chance prior to the onset of nonspecific symptoms.

The treatment of gastrointestinal lymphoma remains controversial. Chemotherapy has been the mainstay of treatment for extranodal lymphomas, while the role of surgery has been less clear (51). In nearly half of previous studies, the combination of surgery and chemotherapy was found to improve overall survival (8, 20, 39, 52–59). However, some studies suggested that the combination of surgery and chemotherapy had no effect on OS or that its impact was uncertain (1, 39, 60–63). Surgical resection is preferred for patients with significant gastrointestinal bleeding, blockage, or perforation (37, 64). In our series, patients with prevalent B-cell pathological types, such as DLBCL, MALT lymphoma, and FL, achieved a high long-term survival rate after surgery, despite the presence of severe complications. However, for T-cell lymphoma and MCL, the postoperative prognosis was poor, regardless of whether patients received selective surgery before chemotherapy or emergency surgery due to severe complications. Therefore, we propose that surgery may improve the survival of some pathological types with relatively good prognoses, but this remains to be conclusively indicated. For certain pathological types, including T-cell lymphoma and MCL, surgery appears to have no discernible influence on prognosis. Further studies with larger sample sizes are needed to evaluate the efficacy of various therapeutic approaches in improving prognosis.

T-cell lymphoma and stages III-IV have previously been shown to indicate a lower survival rate in numerous studies (2, 65–67). In this investigation, we found that “B” symptoms and ulcerative endoscopy type were also associated with a lower survival rate. Contrary to previous studies (2, 7, 17, 18), we did not observe any association between normal LDH levels and prolonged OS, or between the ileocecal region and improved survival. Interestingly, patients with shown factors such as T-cell lymphoma, stages III-IV, “B” symptoms and ulcerative type were often accompanied by abnormal levels of LDH, ADA, HGB and ALB. We believe that these laboratory indicators have the potential to evaluate prognosis; however, additional large-sample randomized controlled trials are necessary to confirm this association.

However, this study has several limitations. First, the sample size of a single center is relatively small, and some results may require further validation through large-scale, multicenter studies. Second, the incidence of PSIL was relatively low, and the study covered a wide range of pathological types, with some types having a small number of cases. Therefore, prognostic judgment based on endoscopic features and laboratory examinations may have certain limitations. Finally, we updated the endoscopic classification system based on previous studies. However, as previous studies mainly focused on a single pathological type of lymphoma, including different types of lymphoma in our study may cause some bias in the number of specific endoscopic features.

In conclusion, this study provides a comprehensive analysis of the clinical and histological characteristics of PSIL, establishing an endoscopic classification and performing a survival analysis. The ileocecal region is the most common site for PSIL. DLBCL and MALT lymphoma were the predominant pathological subtypes. The novel categorization of the five endoscopic subtypes of PSIL developed in this study has potential for use in diagnosis and prognosis. Poor prognosis was associated with stages III-IV, “B” symptoms, T-cell lymphoma, and ulcerative type. The requirement for surgical resection should be determined based on individual patient characteristics. Overall, these findings provide valuable insights into the clinical features and outcomes of PSIL and can help improve the diagnosis, treatment, and prognosis of this disease.

## Data availability statement

The original contributions presented in the study are included in the article/**Supplementary Material**. Further inquiries can be directed to the corresponding author.

## Ethics statement

The studies involving human participants were reviewed and approved by Research Ethics Committee of Shandong University Qilu Hospital. Written informed consent for participation was not required for this study in accordance with the national legislation and the institutional requirements. Written informed consent was not obtained from the individual(s) for the publication of any potentially identifiable images or data included in this article.

## Author contributions

F-YT performed the clinical reviews and data collection, analyzed the data and drafted the manuscript. F-YT and GH reviewed the endoscopic images. SW, WA, L-SA collected and interpreted the data. P-ZW analyzed the data. Y-BY obtained the funding, designed the study, reviewed the endoscopic images and critically revised the manuscript. X-LZ and Y-QL designed the study and critically revised the manuscript. All authors discussed the results and commented on the manuscript. All authors contributed to the article and approved the submitted version.
