# Supplementary material for: Clinical and endoscopic features of primary small bowel lymphoma: a single-center experience from mainland China
Source: Front Oncol. 2023 Jun 16;13:1142133. doi: 10.3389/fonc.2023.1142133 (PMC10313208; doi:10.3389/fonc.2023.1142133)
Supplement: Supplementary file 1 [file Table_1.docx]

Supplementary Material

Clinical and endoscopic features of primary small bowel lymphoma: a single-center experience from mainland China

**Feng-Yu Tian, Jue-Xin Wang, Gang Huang, Wen An, Li-Si Ai, Sui Wang, Pei-Zhu Wang, Yan-Bo Yu^*^, Xiu-Li Zuo, Yan-Qing Li**

*** Correspondence:**

Yan-Bo Yu: Department of Gastroenterology, Qilu Hospital, Shandong University, Jinan, Shandong, P.R. Tel: +86-531-82166012; Fax: +86-531-82166012; China E-mail: yuyanbo2000@126.com

# Supplementary Tables

**Supplementary Table S1 Correlation Between Endoscopic Features and Clinical Manifestations of Primary Small Intestinal Lymphoma**

| **Clinical manifestations** | **Endoscopic features (number of patients)** | | | | | ***P* value** |
| --- | --- | --- | --- | --- | --- | --- |
|  | **Hypertrophic type** | **Exophytic type** | **Follicular/polypoid type** | **Ulcerative type** | **Diffusion type** |  |
| Asymptomatic | 0 | 1 | 6 | 0 | 1 | 0.017 |
| Abdominal pain | 5 | 12 | 6 | 10 | 10 |  |
| Distension | 4 | 3 | 1 | 0 | 2 |  |
| Nausea/vomiting | 2 | 2 | 0 | 1 | 0 |  |
| Diarrhea | 2 | 1 | 0 | 1 | 1 |  |
| Bleeding | 1 | 0 | 4 | 4 | 3 |  |
| "B" symptoms | 1 | 3 | 0 | 3 | 1 |  |

**Supplementary Table S2 Gender and Clinical Characteristics of Primary Small Intestinal Lymphoma**

|  | **Male** | **Female** | ***P* value** |
| --- | --- | --- | --- |
| Age | 54.92±13.92 | 58.14±11.60 | 0.233 |
| Site |  |  |  |
| Duodenum | 8 | 11 | 0.412 |
| Jejunum | 8 | 9 |  |
| Ileum | 10 | 5 |  |
| Ileocecum | 18 | 14 |  |
| Multiple | 8 | 3 |  |
| Pathological Type |  |  |  |
| DLBCL | 23 | 14 | 0.131 |
| MALT | 11 | 16 |  |
| FL | 5 | 6 |  |
| EATL/MEITL | 2 | 3 |  |
| Others | 11 | 3 |  |
| Endoscopic features |  |  |  |
| Hypertrophic type | 8 | 8 | 0.812 |
| Exophytic type | 15 | 8 |  |
| Follicular/polypoid type | 9 | 8 |  |
| Ulcerative type | 11 | 8 |  |
| Diffusion type | 9 | 10 |  |
| Clinical symptoms^1^ |  |  |  |
| Asymptomatic | 3 | 5 | 0.372 |
| Abdominal pain | 23 | 20 |  |
| Distension | 7 | 3 |  |
| Nausea and vomiting | 4 | 1 |  |
| Diarrhea | 3 | 2 |  |
| Hematemesis, hematochezia | 4 | 8 |  |
| "B" symptoms | 6 | 2 |  |
| Laboratory index |  |  |  |
| Hemoglobin (g/L) | 128.00(32.50) | 110.00(38.50) | <0.01 |
| LDH (U/L) | 207.50(90.20) | 193.50(61.30) | 0.258 |
| ALB (g/L) | 38.35(8.35) | 38.40(8.42) | 0.818 |
| ADA (U/L) | 13.00(8.50) | 13.00(7.75) | 0.913 |

^1^ Clinical symptoms are the first symptom or the most severe symptom in the chief complaint.

DLBCL: Diffuse large B-cell lymphoma; MALT: Mucosa-associated lymphoid tissue lymphoma; FL: Follicular lymphoma; EATL/MEITL: Enteropathy-associated T-cell lymphoma/Monomorphic epitheliotropic T cell lymphoma; HGB: Hemoglobin; LDH: Lactate dehydrogenase; ALB: Albumin; ADA: Adenosine deaminase.

**Supplementary Table S3 Age and Clinical Characteristics of Primary Small Intestinal Lymphoma**

|  | **Age** | | ***P* value** |
| --- | --- | --- | --- |
|  | **＜65** | **≥65** |  |
| Pathological Type |  |  |  |
| DLBCL | 24 | 13 | 0.048 |
| MALT | 15 | 12 |  |
| FL | 10 | 1 |  |
| EATL/MEITL | 3 | 2 |  |
| Others | 13 | 1 |  |
| Site |  |  |  |
| Duodenum | 15 | 4 | 0.088 |
| Jejunum | 13 | 4 |  |
| Ileum | 6 | 9 |  |
| Ileocecum | 22 | 10 |  |
| Multiple | 9 | 2 |  |
| Endoscopic features |  |  |  |
| Hypertrophy type | 10 | 6 | 0.463 |
| Exophytic type | 15 | 8 |  |
| Follicular/polypoid type | 15 | 2 |  |
| Ulcerative type | 13 | 6 |  |
| Diffusion type | 12 | 7 |  |
| Clinical symptoms^1^ |  |  |  |
| Asymptomatic | 6 | 2 | 0.294 |
| Abdominal pain | 27 | 16 |  |
| Distension | 5 | 5 |  |
| Nausea and vomiting | 3 | 2 |  |
| Diarrhea | 3 | 2 |  |
| Hematemesis，hematochezia | 11 | 1 |  |
| "B" symptoms | 7 | 1 |  |
| Laboratory index |  |  |  |
| HGB (g/L) | 122.00(33.50) | 118.00(26.50) | 0.970 |
| LDH (U/L) | 204.00(79.00) | 197.00(58.50) | 0.493 |
| ALB (g/L) | 39.00(9.50) | 38.00(5.20) | 0.111 |
| ADA (U/L) | 13.00(8.50) | 12.50(3.75) | 0.744 |

^1^ The clinical symptoms are the first or most severe symptoms mentioned in the chief complaint.

DLBCL: Diffuse large B-cell lymphoma; MALT: Mucosa-associated lymphoid tissue lymphoma; FL: Follicular lymphoma; EATL/MEITL: Enteropathy-associated T-cell lymphoma/Monomorphic epitheliotropic T cell lymphoma; HGB: Hemoglobin; LDH: Lactate dehydrogenase; ALB: Albumin; ADA: Adenosine deaminase.

**Supplementary Table S4 Stage and Clinical Characteristics of Primary Small Intestinal Lymphoma**

|  | **Stage** | | ***P* value** |
| --- | --- | --- | --- |
|  | **I-II** | **III-IV** |  |
| Age | 56.47±13.08 | 56.39±13.59 | 0.909 |
| Gender |  |  |  |
| male | 36 | 14 | 0.509 |
| Female | 32 | 9 |  |
| Histopathologic Type |  |  |  |
| DLBCL | 28 | 8 | 0.372 |
| MALT | 20 | 6 |  |
| FL | 9 | 1 |  |
| T | 8 | 6 |  |
| Other B | 3 | 2 |  |
| Laboratory index |  |  |  |
| HGB (g/L) | 121.0±21.4 | 108.3±27.3 | 0.026 |
| LDH (U/L) | 192.0(52.0) | 235.0(129.0) | <0.01 |
| ALB (g/L) | 39.0(6.9) | 35.5(7.5) | <0.01 |
| ADA (U/L) | 12.0(5.5) | 21.0(20.0) | <0.01 |

DLBCL: Diffuse large B-cell lymphoma; MALT: Mucosa-associated lymphoid tissue lymphoma; FL: Follicular lymphoma; T: T-cell lymphoma; Other B: Mantle cell lymphoma and uncertain subtype B-cell lymphoma; HGB: Hemoglobin; LDH: Lactate dehydrogenase; ALB: Albumin; ADA: Adenosine deaminase

**Supplementary Table S5 Pathology and Clinical Characteristics of Primary Small Intestinal Lymphoma**

|  | **Pathological type** | | ***P* value** |
| --- | --- | --- | --- |
|  | **B** | **T** |  |
| Age | 54.79±11.34 | 56.64±13.28 | 0.625 |
| Site |  |  |  |
| Duodenum | 15 | 4 | 0.067 |
| Jejunum | 11 | 6 |  |
| Ileum | 14 | 1 |  |
| Ileocecum | 30 | 2 |  |
| Multiple | 10 | 1 |  |
| Endoscopic features |  |  |  |
| Hypertrophy type | 15 | 1 | 0.130 |
| Exophytic type | 17 | 6 |  |
| Follicular/polypoid type | 17 | 0 |  |
| Ulcerative type | 15 | 4 |  |
| Diffusion type | 16 | 3 |  |
| Imaging feature(CT)，n(%) |  |  |  |
| Thickening | 40(62.50) | 10(76.92) | 0.500 |
| Occupying | 12(18.75) | 4(30.77) | 0.549 |
| Lymph node enlargement | 44(68.75) | 10(76.92) | 0.799 |
| Clinical symptoms |  |  |  |
| Asymptomatic | 7 | 1 | 0.038 |
| Abdominal pain | 38 | 5 |  |
| Distension | 9 | 1 |  |
| Nausea and vomiting | 5 | 0 |  |
| Diarrhea | 4 | 1 |  |
| Hematemesis，hematochezia | 11 | 1 |  |
| "B" symptoms | 3 | 5 |  |
| Therapy |  |  |  |
| Surgical | 23 | 5 | 0.633 |
| Non-surgical | 36 | 8 |  |
| Conservative management | 15 | 1 |  |
| Laboratory index |  |  |  |
| HGB (g/L) | 126.00(29.70) | 102.50(30.50) | <0.01 |
| LDH (U/L) | 200.00(79.00) | 203.00(41.00) | 0.797 |
| ALB (g/L) | 39.05(6.80) | 32.20(7.45) | <0.01 |
| ADA (U/L) | 12.00(8.00) | 15.00(14.00) | 0.136 |

Conservative management strategies encompass a wide range of interventions, including but not limited to appropriate nutritional support, psychological support, physical therapy, and symptom control medication.

B: B-cell lymphoma; T: T-cell lymphoma; HGB: Hemoglobin; LDH: Lactate dehydrogenase; ALB: Albumin; ADA: Adenosine deaminase
